# Supplementary material for: Polarization of Macrophages in Human Adipose Tissue is Related to the Fatty Acid Spectrum in Membrane Phospholipids
Source: Nutrients. 2019 Dec 18;12(1):8. doi: 10.3390/nu12010008 (PMC7020093; doi:10.3390/nu12010008)
Supplement: Supplementary file 1 [file nutrients-12-00008-s001.zip › Suplementary/Diet questionaire.docx]

**DIETARY QUESTIONNAIRE**

Name: Surname: Date of operation:

**1. Have you substantially changed your currrent diet from that before operation:**

A. YES, in the long term

B. YES, transiently

C. Not, did not change, have not changed

**2. in your family, Do you use for cooking**

1. Only vegetable fats (oil, soft margarines) - 1
2. A combination – both vegetable and animal fat

(butter and lard) - 4

1. Only animal fats - 6

**3. For spreading on bread or rolLs do you use:**

1. Only vegetable fats (oil, soft margarines, mayonNaise - 1
2. A combination – both vegetable fats and animal fat - 2
3. Only animal fats - 3
4. NONE OF THE ABOVE - 0

**4. Fish in diet:**

1. once a week - 1
2. more than once a week - 2
3. rarely - 3
4. ALMOST NEVER - 4

**6. nuts in diet**

1. a lot of - 1
2. occasionally - 2
3. ALMOST NEVER - 3
