# Supplementary material for: Polarization of Macrophages in Human Adipose Tissue is Related to the Fatty Acid Spectrum in Membrane Phospholipids
Source: Nutrients. 2019 Dec 18;12(1):8. doi: 10.3390/nu12010008 (PMC7020093; doi:10.3390/nu12010008)
Supplement: Supplementary file 1 [file nutrients-12-00008-s001.zip › Suplementary/Diet questionaire.pdf]

DIETARY QUESTIONNAIRE

Name: Surname: Date of operation:

1. HAVE YOU SUBSTANTIALLY CHANGED YOUR CURRRENT DIET FROM THAT BEFORE OPERATION:

A. YES, in the long term

B. YES, transiently

C. NOT, did not change, have not changed

2. IN YOUR FAMILY, DO YOU USE FOR COOKING

A. ONLY VEGETABLE FATS (OIL, SOFT MARGARINES) - 1
B. A COMBINATION – BOTH VEGETABLE AND ANIMAL FAT (BUTTER AND LARD) - 4
C. ONLY ANIMAL FATS - 6

3. FOR SPREADING ON BREAD OR ROLLS DO YOU USE:

A. ONLY VEGETABLE FATS (OIL, SOFT MARGARINES, MAYONNAISE - 1
B. A COMBINATION – BOTH VEGETABLE FATS AND ANIMAL FAT - 2
C. ONLY ANIMAL FATS - 3
D. NONE OF THE ABOVE - 0

4. FISH IN DIET:

A. ONCE A WEEK - 1
B. MORE THAN ONCE A WEEK - 2
C. RARELY - 3
D. ALMOST NEVER - 4

6. NUTS IN DIET

A. A LOT OF - 1
B. OCCASIONALLY - 2
C. ALMOST NEVER - 3
