# Supplementary material for: Polarization of Macrophages in Human Adipose Tissue is Related to the Fatty Acid Spectrum in Membrane Phospholipids
Source: Nutrients. 2019 Dec 18;12(1):8. doi: 10.3390/nu12010008 (PMC7020093; doi:10.3390/nu12010008)
Supplement: Supplementary file 1 [file nutrients-12-00008-s001.zip › Suplementary/Table S1.docx]

Table S1: Comparison of FA spectrum in adipocytes and stromal vascular fraction (%)

|  | 16:00 | 16:1 | 18:00 | 18:1n9 | 18:2n6 | 18:3n3 | 20:4n6 | 20:5n3 | 22:6n3 |
| --- | --- | --- | --- | --- | --- | --- | --- | --- | --- |
| SVF % | 21.50 | 1.62 | 19.33 | 28.01 | 12.63 | 1.63 | 4.92 | 0.97 | 2.61 |
| Adipocytes % | 25.62 | 1.78 | 21.85 | 25.44 | 12.70 | 2.24 | 3.06 | 0.59 | 2.06 |
