# Supplementary figures and images for: Polarization of Macrophages in Human Adipose Tissue is Related to the Fatty Acid Spectrum in Membrane Phospholipids
Source: Nutrients. 2019 Dec 18;12(1):8. doi: 10.3390/nu12010008 (PMC7020093; doi:10.3390/nu12010008)

Figure S1: Correlation of the proportion antiinflammatory ATMs to dietary score (n=39; ** p<0.01)


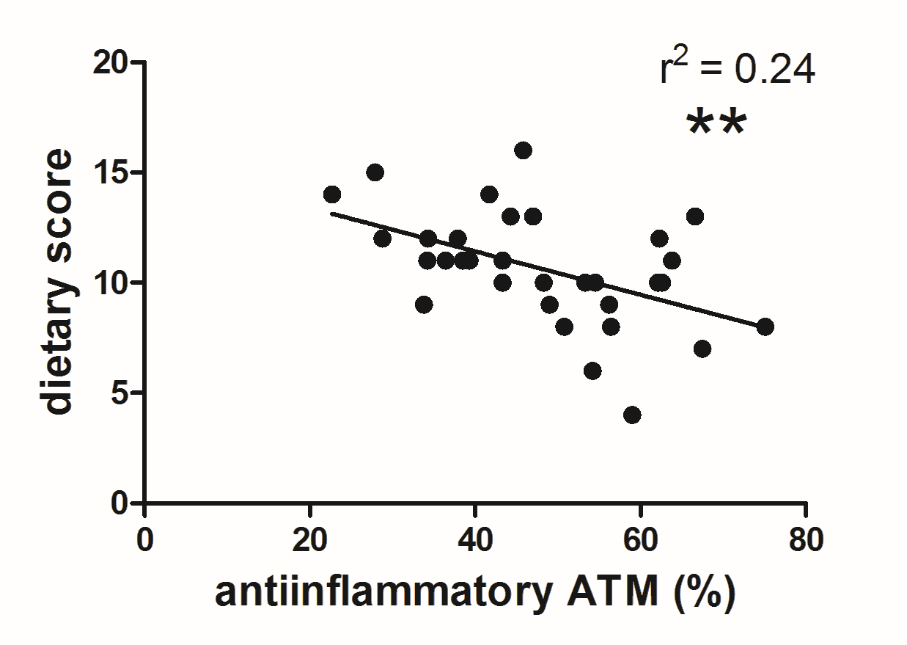

Supplement: Supplementary file 1 [file nutrients-12-00008-s001.zip › Suplementary/Figure S1.docx]
